# S-Adenosylmethionine Inhibits Colorectal Cancer Cell Migration through Mirna-Mediated Targeting of Notch Signaling Pathway †

**DOI:** 10.3390/ijms23147673

**Published:** 2022-07-12

**Authors:** Luigi Borzacchiello, Roberta Veglia Tranchese, Roberta Grillo, Roberta Arpino, Laura Mosca, Giovanna Cacciapuoti, Marina Porcelli

**Affiliations:** Department of Precision Medicine, University of Campania “Luigi Vanvitelli”, Via Luigi De Crecchio 7, 80138 Naples, Italy; luigi.borzacchiello@unicampania.it (L.B.); roberta.vegliatranchese@unicampania.it (R.V.T.); roberta.grillo@unicampania.it (R.G.); roberta.arpino@unicampania.it (R.A.); giovanna.cacciapuoti@unicampania.it (G.C.); marina.porcelli@unicampania.it (M.P.)

**Keywords:** S-Adenosylmethionine, colorectal cancer, breast cancer, Notch, miRNA, metastasis, EMT

## Abstract

Metastasis is a leading cause of mortality and poor prognosis in colorectal cancer (CRC). Thus, the identification of new compounds targeting cell migration represents a major clinical challenge. Recent findings evidenced a central role for dysregulated Notch in CRC and a correlation between Notch overexpression and tumor metastasis. MicroRNAs (miRNAs) have been reported to cross-talk with Notch for its regulation. Therefore, restoring underexpressed miRNAs targeting Notch could represent an encouraging therapeutic approach against CRC. In this context, S-adenosyl-L-methionine (AdoMet), the universal biological methyl donor, being able to modulate the expression of oncogenic miRNAs could act as a potential antimetastatic agent. Here, we showed that AdoMet upregulated the onco-suppressor miRNAs-34a/-34c/-449a and inhibited HCT-116 and Caco-2 CRC cell migration. This effect was associated with reduced expression of migration-/EMT-related protein markers. We also found that, in colorectal and triple-negative breast cancer cells, AdoMet inhibited the expression of Notch gene, which, by luciferase assay, resulted the direct target of miRNAs-34a/-34c/-449a. Gain- and loss-of-function experiments with miRNAs mimics and inhibitors demonstrated that AdoMet exerted its inhibitory effects by upregulating miRNAs-34a/-34c/-449a. Overall, these data highlighted AdoMet as a novel Notch inhibitor and suggested that the antimetastatic effects of AdoMet involve the miRNA-mediated targeting of Notch signaling pathway.

## 1. Introduction

Colorectal cancer (CRC) is the third most diagnosed cancer and the second leading cause of cancer associated mortality worldwide according to a Globocan 2020 survey [1]. Despite advances in early diagnosis and treatments including chemotherapy, immunotherapy, antiangiogenics, and surgical treatment, many of CRC patients still undergo high risks of tumor recurrence and metastasis. Several studies indicated that CRC aggressiveness and potential for metastatic spread are associated with the activation of epithelial-mesenchymal transition (EMT), a process playing a crucial role in driving carcinoma invasion and metastasis [2]. Thus, identifying chemical compounds targeting cancer cell migration is highly advantageous.

CRC is the result of dysregulated cellular pathways that promote inappropriate stem-cell-like phenotype, apoptotic resistance, uncontrolled proliferation, and metastatic spread [3]. Recent studies indicated that Notch signaling activation is responsible for the induction of aggressive phenotypic and functional changes in tumor cells consistent with mesenchymal transformation [4]. Notch signaling is an evolutionarily conserved pathway in multicellular organisms that, through cell-to-cell contacts, influences cell-fate decisions during embryonic and postnatal development and plays a critical role in maintaining the balance between cell proliferation, differentiation, and apoptosis and is also involved in metastasis, angiogenesis, and self-renewal [5]. Dysregulated Notch signaling has been found in a variety of neoplastic diseases, where it plays a complex oncogenic or tumor-suppressive role depending on tissue and cellular context [6]. In addition, the overexpression of Notch signaling has been found to be associated with poor prognosis or poor response to treatment of some solid tumors [5,7]. Therefore, therapeutic strategies have first been developed in the preclinical phase and in early-phase clinical trials to target oncogenic functions of Notch in tumor cells [8]. Recent findings highlighted a central role for abnormal Notch in CRC and indicated a correlation between the overexpression of Notch signaling components and CRC progression and metastasis [9].

Accumulating evidence suggests that microRNAs (miRNAs) play a crucial role in the regulation of genes driving CRC initiation, progression, and metastasis [10]. MiRNAs significantly affect numerous cellular processes, including cell development and differentiation, DNA damage repair, cell death, and intercellular communication. To date, more than 2500 miRNAs have been identified in humans, and nearly a third of human genes closely associated with many physiological processes are regulated by miRNAs [11]. Notably, miRNAs do not need perfect complementarity for target recognition, and therefore, a single miRNA can regulate up to one hundred target genes, showing pleiotropic effects and providing opportunities in the field of cancer therapy [12,13,14,15]. MiRNAs are crucially involved in cancer, acting as oncogenes or tumor suppressors depending on the regulatory effects exerted on the expression of their target genes [16]. Generally, oncogenic miRNAs are overexpressed, while tumor suppressive miRNAs are downregulated or completely lost in tumorigenesis, resulting in enhanced tumor progression, invasion, and metastasis [17]. Several lines of evidence suggest that miRNAs play a crucial role in the regulation of genes driving CRC initiation, progression, and metastasis [10]. Moreover, miRNAs have recently been reported to cross-talk with Notch pathway for its regulation [18]. Therefore, based on the remarkable role exerted by Notch in the promotion of CRC metastasis [4], restoring underexpressed miRNAs that target this signaling pathway could represent a promising therapeutic approach against CRC progression.

The new structures, the potential pharmacological activities, and the few harmful side effects on normal cells make natural compounds and their structural analogues effective tools that have been widely used in different clinical settings. Recent findings have documented that natural compounds exert anti-carcinogenic activities by interfering with the initiation, development, and progression of cancer through regulating epigenetic modifications and affecting various signaling pathways [19]. Beyond targeting protein functions, more and more evidence has demonstrated that natural agents exert antitumor activities by altering miRNA expression, providing a new approach to develop innovative and more efficient anticancer strategies based on synergistic combinatorial therapies [20]. In this context, S-adenosyl-l-methionine (AdoMet), a multitargeted and safe FDA-approved natural compound and the universal biological methyl donor in transmethylation reactions, has emerged, over the past two decades, as a promising anticancer therapeutic agent [21,22]. Recently, the antiproliferative properties of AdoMet and its implication in multiple cellular processes including proliferation, differentiation, cell cycle regulation, and apoptosis in various tumor cells have been thoroughly examined in the literature [21,22,23,24,25,26,27,28,29,30,31], and several findings have highlighted the therapeutical potential of AdoMet as an effective adjuvant to chemotherapeutic agents to be used in combined therapy to overcome drug resistance [28,29,30,31]. More and more evidence has also shown that the epigenetic modulation of miRNAs involved in oncogenic functions represents one of the main mechanisms underlying the anticancer activity of AdoMet. The regulation of miRNA’s expression profile by AdoMet has been recently evaluated in breast and in head and neck cancer cells, suggesting that the ability of this natural compound to inhibit proliferation and cell migration, as well as to induce apoptosis in these tumor cells, is mediated by miRNAs [32,33,34,35].

Growing evidence accumulating in the literature in recent years on the anticancer activity exerted by AdoMet in colon cancer cells highlighted the pleiotropic effects of this eclectic multi-target sulfonium compound, evidencing its ability to overcome 5-FU chemoresistance by targeting multiple pathways such as autophagy, P-gp expression, and NF-kB signaling activation and to influence tumor progression by modulating gene expression [36,37,38].

Here, we demonstrated that AdoMet suppressed CRC progression through the inhibition of EMT and migration of HCT-116 and Caco-2 cells. We found that AdoMet upregulated key tumor-suppressive miRNAs as miRNA34a, miRNA34c, and miRNA449a in these tumor cells and that combined treatment AdoMet/miRNA inhibitors partially reversed the antimigratory effect of AdoMet. Finally, we provided novel evidence that AdoMet-induced inhibition of CRC cell migration involves miRNA-mediated targeting of Notch signaling pathway and that the same mechanism is utilized by AdoMet to inhibit cell migration in MDA-MB-231 and MDA-MB-468 triple negative breast cancer (TNBC) cell lines. The findings highlighted AdoMet as a new Notch inhibitor and a promising candidate for the treatment of Notch-dependent highly invasive cancers such as CRC and TNBC.

## 2. Results

### 2.1. AdoMet Upregulated miR-34a, miR-34c, and miR-449a Expression in HCT-116 and Caco-2 CRC Cell Lines

MiR-34a, miR-34c, and miR-449a, belonging to the miRNA-34/449 superfamily, are downregulated in many types of human cancers, including CRC, and play critical roles in tumor development and progression [39,40,41]. The involvement of miRNA-34/449 superfamily in the regulation of oncogenic pathways such as cell proliferation, metastasis, and apoptosis, proposes their potential role as tumor suppressors [41,42,43]. Recently, AdoMet-induced modulation of miR-34c and miR-449a expression has been evaluated in MDA-MB-231 and MDA-MB-468 breast cancer cell lines [33], providing evidence that the inhibition exerted by AdoMet on TNBC cell migration is mediated by AdoMet-induced upregulation of these tumor suppressor miRNAs.

**Figure 1:**
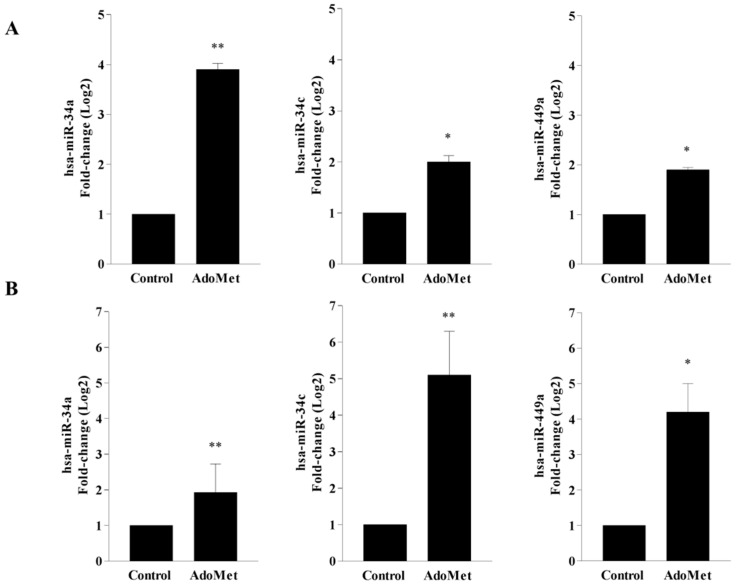
Effect of AdoMet on miR-34a, miR-34c, and miR-449a expression in CRC cells. The relative expression of miR-34a, miR-34c, and miR-449a in HCT-116 (**A**) and Caco-2 (**B**) cells treated with 500 μM AdoMet for 48 h was analyzed by qRT-PCR, following normalization with U6 endogenous control. The analysis was carried out by triplicate determination of at least 3 separate experiments. The results are expressed as fold-change (Log_2_) ± SD. The means were compared using Student’s *t* test, * *p* < 0.05, ** *p* < 0.01.

To gain new information into the molecular mechanisms underlying AdoMet’s antitumor activity in CRC cells and to confirm the ability of AdoMet to act as epigenetic regulator of miRNAs, the expression profile of miR-34a, miR-34c, and miR-449a was analyzed in HCT-116 and Caco-2 cells by quantitative real-time PCR (qRT-PCR) analysis with pre-designed probe-primer sets, after cell treatment with 500 µM AdoMet (Figure 1). The results achieved showed that after 48 h, the relative expression of the three miRNAs appeared remarkably upregulated by AdoMet in both CRC cell lines when compared to untreated cells with fold-change values of 3.9-fold and 1.9-fold for miR-34a, 2.0-fold and 5.2-fold for miR-34c, and 1.9-fold and 4.2-fold for miR-449a, in HCT-116 (Figure 1A) and Caco-2 (Figure 1B) cells, respectively. The ability to reprogram the expression of miRNA 34/449 superfamily in HCT-116 and Caco-2 cells, in good agreement with the results obtained in MDA-MB-231 and MDA-MB-468 cells [33], is indicative of a generalized miRNA regulation mechanism exerted by AdoMet in highly invasive cancers such as CRC and TNBC.

### 2.2. miR-34a, miR-34c, and miR-449a Mediated the AdoMet-Induced Inhibition of HCT-116 and Caco-2 Cell Migration

**Figure 2:**
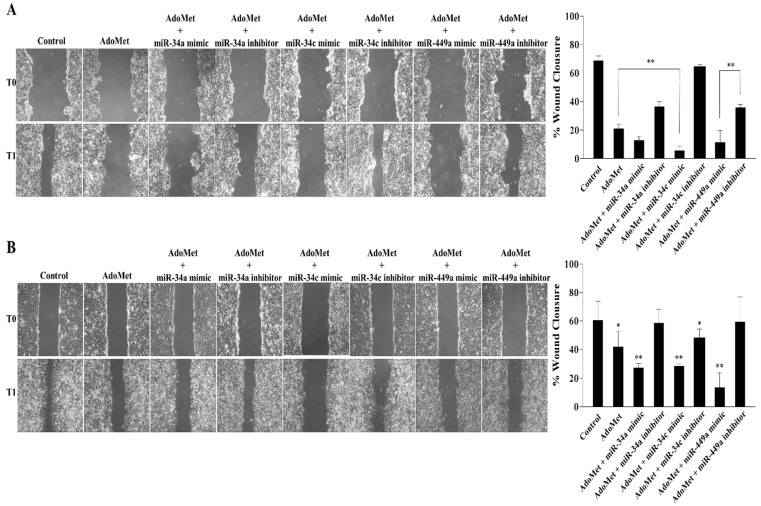
Effect of AdoMet alone and in combination with miR-34a, miR-34c, and miR-449a mimics or inhibitors on CRC cell migration. Confluent monolayers of HCT-116 (**A**) and Caco-2 (**B**) cells treated or not (Control) with 500 μM AdoMet alone or in combination with miR-34a, miR-34c, and miR-449a mimics or inhibitors for 48 h, were scratched with a micropipette tip and snapshot pictures were captured by microscope to check for wound closure. Pictures of the wounds corresponding to time zero (T0) and 24 h (T1) from the scrape in both cell lines are reported. Histograms show the quantification of wound area calculated as a percentage of the control using ImageJ software 1.48v (U.S. National Institutes of Health, Bethesda, MD, USA). Data represent the average of three independent experiments. The means and SD are shown. The means were compared using analysis of variance (ANOVA) plus Bonferroni’s *t*-test. * *p* < 0.05, ** *p* < 0.01 versus untreated cells (Control).

In order to evaluate the involvement of AdoMet-induced miRNA-34/449 superfamily up-regulation in the migration process of HCT-116 and Caco-2 cells, we investigated the effect of AdoMet, alone or in combination with miR-34a, miR-34c, and miR-449a mimics or inhibitors by wound healing assay monitored for 24 h (Figure 2). In both cell lines, we evidenced that compared to untreated cells, the treatment with 500 μM AdoMet for 48 h inhibited CRC cell motility causing approximately 21.1% and 42.0% wound closure in HCT-116 (Figure 2A) and Caco-2 (Figure 2B) cells, respectively. The inhibitory effect of AdoMet was significantly increased following the combined treatments with the sulfonium compound and miR-34a, miR-34c, and miR-449a, leading to wound closure values of about 12.1% and 27.3% for miR-34a, 5.6% and 28.5% for miR-34c and 11.5% and 13.5% for miR-449a in HCT-116 (Figure 2A) and Caco-2 (Figure 2B) cells, respectively, strongly indicating the role of miR-34a, miR-34c, and miR-449a as mediators of the process. Confirming evidence of these results came from loss-of-function experiments showing that treatment with AdoMet in combination with miR-34a, miR-34c, and miR-449a inhibitors reverted the miRNAs- and AdoMet-induced cellular effects and restored the migratory capacity of either HCT-116 (Figure 2A) and Caco-2 (Figure 2B) cells, as revealed by the wound size. Notably, the inhibitory effect exerted by miR-34a, miR-34c, and miR-449a on CRC cell migration is in line with other literature reports on the antimetastatic effects exerted by these miRNAs in various tumors [43,44,45]. Overall, these data indicated that epigenetic regulation of miRNA-34/449 superfamily by AdoMet played a crucial role in regulating HCT-116 and Caco-2 cell motility and represented the likely mechanism underlying AdoMet-induced inhibition of CRC cell migration.

### 2.3. miR-34a, miR-34c, and miR-449a Mediated AdoMet-Induced Inhibition of Migration- and EMT-Related Protein Expression in HCT-116 and Caco-2 Cells

**Figure 3:**
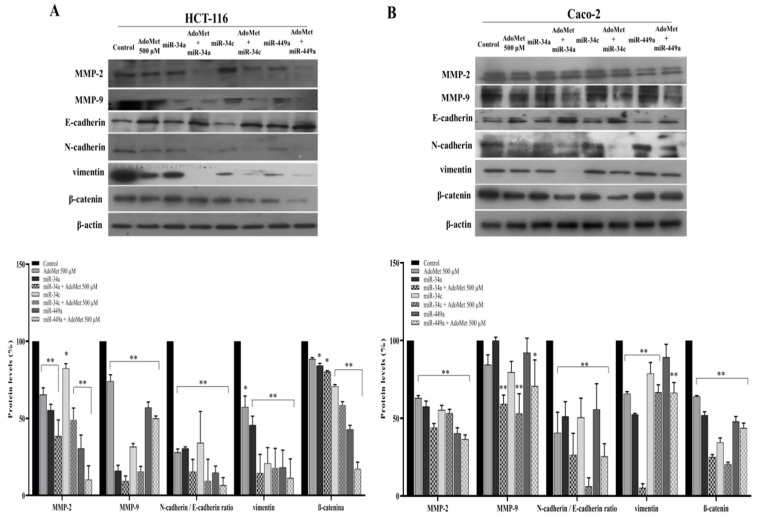
Effect of AdoMet alone and in combination with miR-34a, miR-34c, and miR-449a mimics on the levels of migration- and EMT-related proteins in CRC cells. Cells were transfected with 100 nM miR-34a, miR-34c, and miR-449a in the presence or not (control) of 500 μM AdoMet for 48 h. The expression levels of MMP2, MMP9, E-cadherin, N-cadherin, vimentin, and β-catenin, were detected by Western blot using the total cell lysate of HCT-116 (**A**) and Caco-2 (**B**) cells. The densitometric analysis was reported as the percentage of protein expression of untreated control (100%). For the equal loading of proteins in the lanes, β-actin was used as a standard. The images are representative of three immunoblotting analyses obtained from at least three independent experiments. The means were compared using analysis of variance (ANOVA) plus Bonferroni’s *t*-test. * *p* < 0.05, ** *p* < 0.01 versus untreated cells (Control). Uncropped images of Western blots are reported in Figures S1 and S2.

To confirm the results of the wound healing assay and to investigate whether the epigenetic regulation of miRNAs by AdoMet was involved in the inhibition of cell migration-associated EMT process, we analyzed by Western blot the effect of AdoMet and miR-34a, miR-34c, and miR-449a, alone and in combination, on the expression levels of the main markers characterizing cell migration and EMT in HCT-116 and Caco-2 cells. First, we examined metalloproteinase-2 (MMP-2) and metalloproteinase-9 (MMP-9), key proteolytic enzymes involved in the degradation of the basement membrane and extracellular matrix [46] whose overexpression has been recently correlated with poor survival outcome in CRC patients [47]. We found that AdoMet and miR-34a, miR-34c, and miR-449a individually reduced the expression of MMP-2 and MMP-9 in HCT-116 (Figure 3A) and Caco-2 (Figure 3B) cells as compared to control and that their combined treatment was more effective than single treatment indicating a functional relationship between AdoMet and these miRNAs and suggesting that the antimigratory properties of AdoMet in CRC cells are mediated by the upregulation of tumor suppressors miR-34a, miR-34c, and miR-449a.

In invasive tumors, the upregulation of the mesenchymal marker N-cadherin and downregulation of the epithelial marker E-cadherin represent the hallmark of migratory and invasive traits during EMT [48]. We therefore analyzed the expression patterns of EMT-associated proteins, including E-cadherin, N-cadherin, and vimentin. We found that, after single treatment of HCT-116 (Figure 3A) and Caco-2 (Figure 3B) cells with AdoMet or with miR-34a, miR-34c, and miR-449a, the intensity of E-cadherin protein band compared to untreated cells became more evident in treated cells, while N-cadherin behaved in the opposite way, resulting in an N- to E-cadherin switch [48] indicative of AdoMet-induced inhibition of EMT. The enhanced effect observed in both CRC cell lines following combined AdoMet/miRNAs treatment clearly indicated that AdoMet-dependent upregulation of miR-34a, miR-34c, and miR-449a is involved in the AdoMet-induced transition from N- to E-cadherin expression, in turn, responsible for the suppression of cell migration. We then evaluated vimentin, the major cytoskeletal component of motile mesenchymal cells including metastatic tumor cells of epithelial origin [49]. Additionally, in this case, a strong decrease in the levels of this protein was observable following either single and, more markedly, combined treatment with AdoMet and miR-34a, miR-34c, and miR-449a, furnishing evidence that the modulation of vimentin expression by AdoMet is dependent on these miRNAs.

Finally, we examined β-catenin, the most important mediator of the canonical Wnt signaling pathway, whose dysregulation has been recently demonstrated to play a primary role in driving colon cancer cell growth, invasion, and survival [50]. The results showed, in comparison with the control, a downregulation of β-catenin in HCT-116 (Figure 3A) and Caco-2 (Figure 3B) cells treated with AdoMet or with miR-34a, miR-34c, and miR-449a, which became more evident following a combined AdoMet/miRNA treatment, thus providing evidence that AdoMet negatively regulates β-catenin signaling through modulating miR-34a, miR-34c, and miR-449a expression.

In conclusion, our findings indicated that the combined treatment of AdoMet and miR-34a, miR-34c, and miR-449a was more effective compared to the single agents in inhibiting the main cell migration- and EMT-related protein markers, further confirming the crucial role of these tumor suppressive miRNAs as mediators of the antimigratory and antimetastatic properties of AdoMet in CRC cells.

### 2.4. Notch Was Directly Targeted by miR-34a, miR 34c, and miR 449a in Colorectal HCT-116 and Caco-2 and in Triple Negative MDA-MB-231 and MDA-MB-468 Breast Cancer Cells

**Figure 4:**
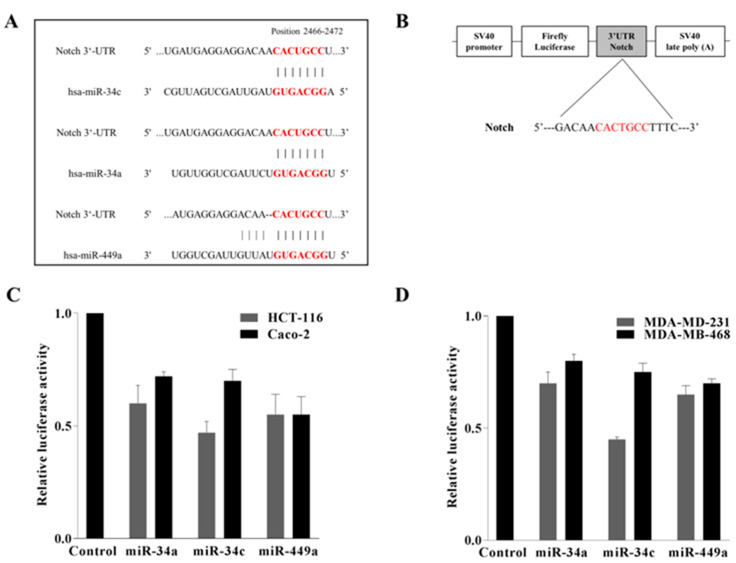
AdoMet-upregulated miR-34a, miR-34c, and miR-449a directly bind to 3′-UTR of Notch in CRC and TNBC cells. Alignments of miR-34a, miR-34c, and miR-449a with Notch 3′-UTR obtained from miRNA-mRNA integration analysis using the microRNA target prediction software TargetScan (**A**). Schematic diagram of luciferase reporter plasmid containing 3′-UTR target sequence of Notch for miRNAs (**B**). The relative luciferase activity was determined using co-transfected CRC HCT-116 and Caco-2 (**C**) and TNBC MDA-MB-231 and MDA-MB-468 (**D**) cells with miR-34a, miR-34c, and miR-449a mimics and the corresponding luciferase reporter plasmid. Each sample was run in triplicate. The means were compared using analysis of variance (ANOVA) plus Bonferroni’s *t*-test. Error bars show mean ± SD.

To unravel the molecular mechanisms of how AdoMet suppresses cell migration and EMT in HCT-116 and Caco-2 CRC cells, the downstream mediators or effectors of AdoMet/miR-34a/34c/449a cascade were predicted and analyzed by two distinct computational algorithms, available online in silico tools, TargetScan 7.1 [51] and miRBase software [52]. Among the putative targets of the miR34/449 superfamily, we have identified and selected Notch gene for further studies on in vitro cell lines, since, in CRC, as well documented in the literature, Notch signaling is dysregulated, and Notch overexpression has been correlated with poor survival, cancer stem cell-like phenotype, EMT, and metastasis resulting in tumor progression [4,5,6,7,8,9,53]. As indicated in Figure 4A, showing the results of the miRNAs/Notch alignments, the 3′-UTR of Notch mRNA contains a perfectly complementary binding site for the seed region of miR-34a/c and miR-449a.

To validate the binding prediction and to find out whether miR-34a/c and miR-449a were able to establish a direct interaction with Notch mRNA, a dual-luciferase reporter assay was performed. The 3′-UTR of Notch gene containing the putative miRNAs binding sites was cloned downstream of luciferase reporter vector to generate the construct shown in Figure 4B. HCT-116 and Caco-2 cells were co-transfected with 3′-UTR reporter construct of Notch along with miR-34a/c or miR-449a mimics prior to cell harvest and luciferase assay. As expected, co-transfected miRNA mimics substantially reduced in both cell lines the luciferase activity of 3′-UTR reporter compared to the control cells (Figure 4C), indicating that the miRNAs of interest target Notch, leading to its downregulation. Notably, the transfection of cells with a luciferase reporter construct carrying mutation in the 3′-UTR binding region abrogated the repression of luciferase expression exerted by miRNAs, confirming the specificity of the interaction (data not shown). In addition, based on the finding of our previous study demonstrating that miR-34c and miR-449a mediated the AdoMet-induced inhibition of MDA-MB-231 and MDA-MB-468 cell migration [33], we decided to carry out the same experiments also in these TNBC cell lines. Figure 4D shows that the results obtained are indicative of a functional interaction between miRNAs and their target site at Notch-3′-UTR, confirming the results obtained in CRC cells and demonstrating that Notch represents the target of miR34/449 superfamily in highly invasive cancer cells such as CRC and TNBC cells.

### 2.5. AdoMet Inhibited Notch Expression in CRC and TNBC Cells through miR-34a, miR-34c, and miR-449a

**Figure 5:**
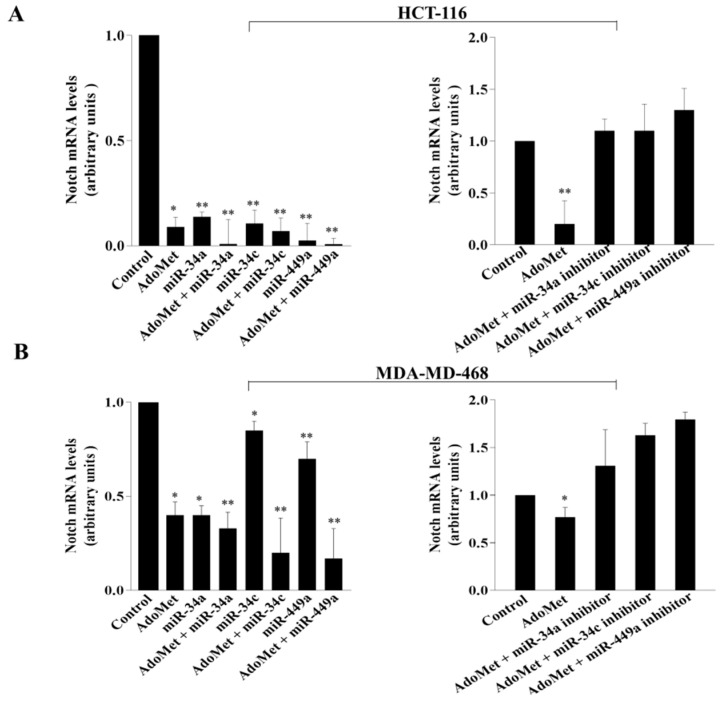
Effect of AdoMet and miR-34a, miR-34c, and miR-449a on Notch mRNA levels in CRC and TNBC cells. HCT-116 (**A**) and MDA-MB-468 (**B**) cells were transfected with miR-34a, miR-34c, and miR-449a mimics (**left**) and inhibitors (**right**) in the presence or not (Control) of 500 μM AdoMet for 48 h. Total-RNA of HCT-116 and MDA-MB-468 cells was extracted, and cDNA was synthesized by qRT-PCR to analyze the transcriptional level of the predicted target. The graphs show the fold-change of Notch in the different experimental conditions normalized to GAPDH mRNA and compared to untreated cells. Data represent the average of three independent experiments. The means were compared using analysis of variance (ANOVA) plus Bonferroni’s *t*-test. Error bars show mean ± SD. * *p* < 0.05, ** *p* < 0.01 versus control untreated cells.

**Figure 6:**
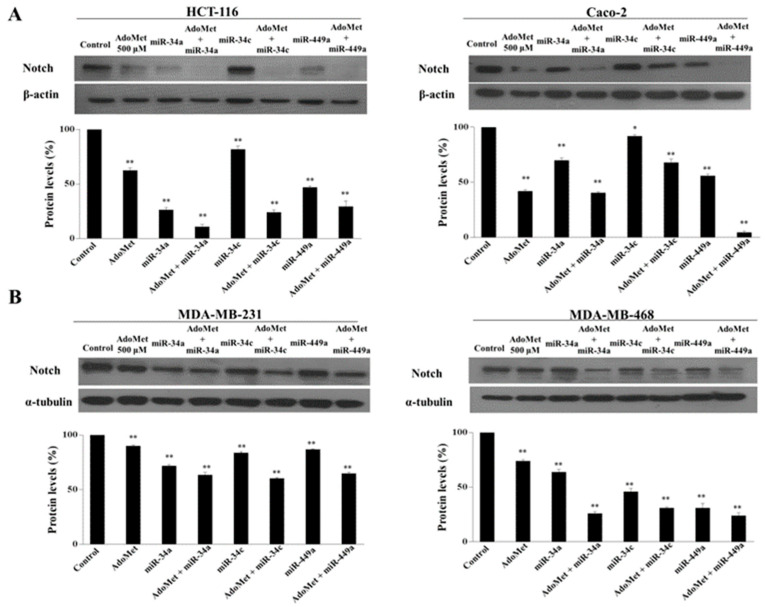
Effect of AdoMet and miR-34a, miR-34c, and miR-449a mimics on Notch protein levels in CRC and TNBC cells. CRC (**A**) and TNBC (**B**) cells were transfected with miR-34a, miR-34c, and miR-449a mimics, in the presence or not (Control), of 500 μM AdoMet for 48 h. Then, cell lysates were subjected to SDS-PAGE, incubated with antibodies against the indicated proteins and analyzed by Western blot. Housekeeping proteins, β-actin and α-tubulin, were used as a loading control. The densitometric analysis was reported as a percentage of protein expression of untreated control (100%). Data represent the average of three independent experiments. The means were compared using analysis of variance (ANOVA) plus Bonferroni’s *t*-test. Error bars show mean ± SD. * *p* < 0.05, ** *p* < 0.01 versus control untreated cells. Uncropped images of Western blots are reported in Figure S3.

To investigate the functional relevance of miRNAs/Notch-3′-UTR interaction in colorectal and in breast cancer cells and to explore whether AdoMet might be able to target Notch expression through miRNAs upregulation, CRC and TNBC cells were transfected with miR-34a, miR-34c, and miR-449a mimics in the presence and in absence of 500 μM AdoMet and Notch expression was then analyzed by RT-qPCR and Western blot. The results indicated that after treatment with AdoMet and miRNA mimics alone, a reduced amount of Notch mRNA (Figure 5A,B, left) and its encoded protein (Figure 6A,B) was detected in both cancer cell types, as compared to the control, demonstrating that AdoMet and miRNAs individually suppressed Notch expression at both translational and transcriptional levels and highlighting Notch as an important target of AdoMet and miR-34a/c/449a superfamily in these tumor cell lines. Interestingly, AdoMet/miRNA mimics co-treatment significantly potentiated the effect of AdoMet (Figure 5A,B left and Figure 6A,B), providing evidence that AdoMet negatively regulated Notch expression through miR-34a, miR-34c, and miR-449a. Notably, loss-of-function studies carried out in HCT-116 cells evidenced that transfection with miRNAs inhibitors was able to revert the inhibitory effect of AdoMet and to restore the levels of Notch mRNA (Figure 5A,B right), further confirming that miRNAs are important mediators of AdoMet-induced inhibition of Notch expression. Taken together, these data highlighted AdoMet as a novel Notch inhibitor and strongly suggested that the antimetastatic effects exerted by AdoMet in colorectal and breast cancer cells are probably mediated by miRNA 34a-34c-449a/Notch axis.

## 3. Discussion

The metastatic spread of cancer cells is a leading cause of mortality in CRC patients. Many studies have pointed out that tumor cell migration and invasion are prerequisites for subsequent metastasis to distant organs and that activation of EMT, a process in which epithelial cells lose their adhesions and gain a mesenchymal migratory and aggressive phenotype, representing the early and crucial step of cancer metastasis and playing a major role in CRC progression [2]. Therefore, it is important to elaborate therapeutic approaches able to target metastasis and its preliminary stages such as cell migration and EMT. Although the pathogenesis of CRC has not been fully elucidated, accumulating evidence has indicated that the dysregulation of miRNAs contributes to the metastatic process and influences CRC development. Thus, restoring the expression of underexpressed tumor suppressive miRNAs is considered a promising and advantageous strategy to control cancer metastasis and to improve the outcome in CRC patients [54].

AdoMet, identified in recent decades as a potent anticancer molecule, is one of the most studied epigenetic regulators. It plays a primary role in cellular metabolism and represents the major methyl donor required in numerous methylation reactions [55,56,57]. Notably, AdoMet has been found to regulate the expression of miRNAs and lncRNAs through epigenetic mechanisms [35]. Recently, the effect of AdoMet on miRNA expression profile has been evaluated in breast [32,33] and in head and neck cancer [34], providing evidence that the ability of AdoMet to inhibit cell proliferation and migration, as well as to induce apoptosis in these tumor cells, is mediated by miRNAs.

In the present study, we demonstrated that AdoMet exhibited antimetastatic activity in CRC cells and provided experimental evidence on the underlying mechanism. The novel finding of this work indicated that AdoMet suppressed EMT and the migration of HCT-116 and Caco-2 CRC cells by activating the expression of miR-34a/c/miR-449a, which directly targeted the Notch gene, leading to its post-transcriptional repression. Comparable results obtained by expanding the study to MDA-MB-231 and MDA-MB-468 cell lines allow us to propose this mechanism as a general strategy utilized by AdoMet to target EMT and cell migration in invasive cancers such as CRC and TNBC (Table S1).

In agreement with the findings previously obtained by our laboratory in the highly invasive breast cancer cells MDA-MB-231 and MDA-MB-468, we found that AdoMet was able to up-regulate the expression of Mir34a/c and miR-449a in HCT-116 and Caco-2 cells, evidencing the ability of the sulfonium compound to reprogram non-coding RNA expression in CRC cells.

The miR-34/449 family is conserved in mammals and includes six homologous genes, miR-34a, miR-34b, miR-34c, miR-449a, miR-449b, and miR-449c, located at three genomic loci. The miR-34/449 family is widely distributed in vertebrate organisms and is important for the regulation of virus–host interactions by modulating immune responses and virus replication [58]. In mammals, miR-34 includes three miRNAs with miR-34a having its own transcript located at chromosome 1p36.22, whereas miR-34b and miR-34c share a primary transcript located at chromosome 11q23.1 [43]. The seed sequence of miR-34a and miR-34c is identical, while miR-34b is slightly different. The mature miR-34a sequence shares 86% and 82% homology with miR-34b and miR-34c, respectively, suggesting that miR-34s could recognize similar mRNA targets and thus could be functionally redundant [59]. Notably, miR-34s show p53-responsive elements in their promoter regions and have been identified as one of the first p53-regulated miRNAs [60]. Converging evidence demonstrated that the overexpression of miR-34s triggers various p53-downstream effects in a context-dependent manner, highlighting these miRNAs as important mediators of tumor-suppressive activities of p53 [61]. MiR-34s play a crucial role in repressing tumor progression by involving in EMT via p53, EMT-transcription factors, and some important signaling pathways, including Notch and Wnt/β-catenin [59]. Consistently with their suppressive role in tumorigenesis, miR-34s are frequently expressed at reduced levels in a broad range of tumors due to aberrant transcriptional regulation, genomic deletions, and promoter hypermethylation [62]. Epigenetic silencing of miR-34s is associated with the proliferation, migration, and invasion of CRC [63]. In analogy with miR-34s, miR-449a is a member of a cluster (miR-449a, miR-449b, and miR-449c) characterized by sequences and secondary structures similar to those of miR-34 members. MiR-449a is considered a tumor-suppressive miRNA, and its downregulated expression has been associated with advanced clinical stage and the poor histological differentiation of CRC [40,64]. Recently, it has been reported that the upregulation of autophagy-dependent miR-449a suppresses CRC tumorigenesis both in vitro and in vivo [65]. Based on these findings, the ability to reactivate the expression of the tumor-suppressive miR-34a/c/miR-449a, thereby restoring their levels in CRC cells, makes AdoMet a potential therapeutic agent for colorectal cancer.

We found that AdoMet caused a decrease in the migratory rate of HCT-116 and Caco-2 cells indicative of its ability to lower the aggressiveness of these cancer cells and potentially reduce their metastatic power.

MMPs have a key function in extracellular matrix remodeling and degradation and play roles in all stages of carcinogenesis [46,47]. Among MMPs, MMP-2 and MMP-9 have drawn much attention for their implication in tumor invasion and metastasis. These enzymes have a unique ability to break down type IV collagen, which is a major component of the basement membrane. Elevated expression of MMP-2/9 has been associated with increased metastatic potential in many tumor cells, including CRC [46]. Moreover, many studies revealed a correlation between increased MMP-2/9 expression and worst outcome of CRC suggesting that the control of the expression and/or the activity of these proteolytic enzymes may offer a new therapeutic opportunity for treating this highly invasive cancer [66]. In line with this view, we found that the decrease in cell migration induced by AdoMet in HCT-116 and Caco-2 cells was associated with decreased protein levels of MMP-2 and MMP-9.

The switch from E-cadherin to N-cadherin expression is considered an important marker of ongoing EMT and has been shown to promote cancer cell motility and invasion [48]. Furthermore, tumor cells subjected to EMT significantly modify the composition of cytoskeletal intermediate filaments with the repression of keratin and expression of vimentin that is believed to be responsible for the adoption of a mesenchymal shape and increased motility and is regarded as the main and canonical marker of EMT [67]. Vimentin is highly expressed in CRC cells, where it plays a critical role in metastasis and prognosis [68]. Notably, our results showed that in HCT-116 and Caco-2 cells, AdoMet significantly decreased vimentin levels and promoted the switch from N- to-E-cadherin expression, as evidenced by comparing the intensities of the corresponding protein bands in AdoMet-treated and untreated cells highlighting the ability of AdoMet to slow down CRC cell migration by inhibiting the transition from the epithelial to the mesenchymal state.

Wnt/β-catenin signaling is one of the most frequently dysregulated pathways in CRC [69]. Aberrant activation of this signaling causes the accumulation of β-catenin in the nucleus and promotes the transcription of many Wnt target genes involved in the initiation, progression, metastasis, drug resistance, and immune evasion of cancer [70]. Notably, β-catenin has been recently recognized as a direct target of miR-34b in human colon cancer cells and it has been reported that miR-34b may inhibit migration and invasion of Caco-2 cells by regulating Wnt/β-catenin signaling [71]. Several inhibitors of Wnt/β-catenin signaling pathway have been developed for CRC treatment [71]. Interestingly, many of these inhibitors currently under investigation include natural compounds, drugs, small molecules, and biological agents [72]. In line with this evidence, we found that AdoMet caused a decrease in β-catenin level in HCT-116 and Caco-2 cells, allowing us to consider this physiological sulfonium compound a promising agent for the selective targeting of Wnt/β-catenin signaling pathway.

The activation of Notch signaling has been shown to play a key role in the induction of the aggressive and metastatic phenotype of CRC cells [73]. In recent literature, a complex network of mutual interconnections between miR-34s and Notch signaling has been reported and discussed indicating that this interplay is implicated in cancer initiation/progression, metastasis, and chemoresistance [73]. In ovarian cancer, the overexpression of miR-34 mimic induced cell death and autophagy through downregulating Notch1, whereas Notch1 transfection reverted anti-proliferative effects of miR-34 [74]. In CRC, it has been reported that miR-34 inversely correlated with metastasis and that its overexpression suppressed cell invasiveness and migration by targeting Notch1 and JAG1 [75]. Moreover, a natural compound, genistein, inhibited cell growth and induced apoptosis in pancreatic cancer cells through the upregulation of miR-34a, leading to decreased Notch1 [76]. Regarding miR-449a, it has been recently reported that its overexpression in vitro led to the silencing of genes associated with Notch signaling in prostate cancer cells [77]. Consistent with these reports, we identified Notch as a direct target of miR-34a/c/miR-449a and provided new evidence that AdoMet was able to inhibit Notch expression through upregulation of miR-34a/c/miR-449a.

Members of miR-34/miR-449 family sharing similar sequences allow to expect similar biological roles and target genes [78]. Notably, we found that: (i) the overexpression of miR-34a/c/miR-449a induced by AdoMet or through transfecting the cells with the corresponding miRNA mimics, caused comparable inhibitory effects on the migratory power of HCT-116 and Caco-2 cells and on the expression levels of relevant proteins associated with cell migration and EMT such as MMP-2, MMP-9, E-cadherin, N-cadherin, vimentin, β-catenin, and Notch; (ii) the ectopic expression of miR-34a/c/miR-449a in cells transfected with miRNA mimics significantly enhanced the inhibitory effects induced by AdoMet resulting in a gain-of-function; (iii) the loss-of-function studies performed by transfecting CRC cells with miRNA inhibitors reverted the AdoMet-induced inhibition on cell migration and on the levels of Notch mRNA and its encoded protein. Consistent with these findings, the key primary step of the antimetastatic activity of AdoMet in HCT-116 and Caco-2 cells is represented by the AdoMet-induced upregulation of miR-34a/c/miR-449a. In fact, transfection with miRNA inhibitors significantly diminished the observed inhibitory effects of AdoMet on EMT and cell migration. In turn, miR-34a/c/miR-449a act as crucial players downstream of AdoMet through the directly targeting of Notch signaling pathway.

## 4. Materials and Methods

### 4.1. Materials

Tissue culture dishes were purchased from Corning (Corning, NY, USA). Bovine serum albumin (BSA), fetal bovine serum (FBS), Dulbecco’s modified Eagle’s medium (DMEM), phosphate-buffered saline (PBS), and trypsin-EDTA were obtained from Gibco (Grand Island, NY, USA). AdoMet was provided from New England Biolabs, prepared in a solution of 5 mM H_2_SO_4_ and 10% ethanol, filtered, and stored at −20 °C until use. Opti-minimal essential medium (Opti-MEM), mirVANA PARIS Kit, Lipofectamine 2000, TaqMan-MiRNA Reverse Transcription Kit, High-capacity cDNA reverse transcription kit, TaqMan Universal PCR Master Mix, small-nuclear-U6, miRNA-34a, miRNA-34c and miRNA-449a mimics and inhibitors, SYBR™ Green PCR Master Mix and custom DNA Oligos: neurogenic locus notch homolog protein (Notch), glyceraldehyde 3-phosphate dehydrogenase (GAPDH) were obtained from Thermo Fisher Scientific (Waltham, MA, USA). The pEZX-MT06 plasmid containing 3′-UTR of Notch were obtained from GeneCopoeia (Rockville, MD, USA), and Dual-Luciferase Reporter Assay System was purchased from Promega (Madison, WI, USA). Radioimmunoprecipitation assay buffer (RIPA buffer) was purchased from Sigma-Aldrich (St. Louis, MO, USA). Monoclonal antibodies (mAbs) to β-actin (#3700, dilution: 1:5000), α-tubulin (#2125, dilution: 1:5000), MMP9 (#13667, dilution: 1:1000), vimentin (#5741, dilution: 1:1000), β-catenin (#8480, dilution: 1:1000), N-cadherin (#13116, dilution: 1:1000), E-cadherin (#14472, dilution: 1:1000), and Notch (#5732, dilution: 1:1000) and polyclonal antibodies (pAbs) to MMP2 (#4022, dilution: 1:1000) were purchased from Cell Signaling Technology (Danvers, MA, USA). Horseradish peroxidase (HRP)-conjugated goat anti-mouse (GxMu-003-DHRPX) and HRP-conjugated goat anti-rabbit (GtxRb-003-DHRPX) secondary antibodies were obtained from ImmunoReagents Inc. (Raleigh, NC, USA). All buffers and solutions were prepared with ultra-high-quality water. All reagents were of the purest commercial grade.

### 4.2. Cell Cultures and Treatments

CRC cell lines HCT-116 and Caco-2 and TNBC cell lines MDA-MB-468 and MDA-MB-231 provided from the American Type Culture Collection (ATCC, Manassas, VA, USA) were cultured at 37 °C in a 5% CO_2_ humidified atmosphere and grown in DMEM supplemented with 10% FBS, 2 mM L-glutamine and 50 U/mL penicillin-streptomycin. Typically, subconfluent cells were seeded in 6-well plates at a density of 2.5 × 10^5^ cells/well and 1.5 × 10^5^ cells/well for CRC and TNBC cells, respectively, to achieve 80% confluence. After 24 h, the cells were treated with 10% FBS fresh medium containing 500 µM AdoMet for 48 h. Subsequently, fluctuating cells were recovered from culture medium by centrifugation, whereas adherent cells were collected by trypsinization.

### 4.3. Cell Transfections

CRC and TNBC cells, at 80% confluence, were transfected with 100 nM miR-34a, miR-34c and miR-449a mimics or inhibitors diluted in Opti-MEM medium added or not (Control) with 500 μM AdoMet, using Lipofectamine 2000 according to the manufacturer’s protocol. Lipofectamine was also used alone as a negative control. After 48 h from transfection, the cells were harvested and then processed to carry out the appropriate analyses.

### 4.4. qRT-PCR for miRNAs Detection and mRNA Expression 

Total RNA from cultured HCT-116 and Caco-2 cells and from MDA-MB-468 cells transfected with 100 nM miR-34a, miR-34c and miR-449a mimics, treated or not with AdoMet 500 µM for 48 h, was purified using the mirVANA PARIS kit, according to the manufacturer’s protocol. RNA concentration was determined using a NanoDrop 1000 spectrophotometer (Thermo Fisher Scientific, Waltham, MA, USA). Subsequently, using the TaqMan MiRNA Reverse Transcription Kit or high-capacity cDNA reverse transcription kit, single-stranded cDNA was synthesized from total RNA samples.

The expression of individual miRNAs was determined using pre-designed probe-primer sets from Life Technologies and TaqMan Universal PCR Master Mix by quantitative real-time PCR (qRT-PCR) performed on a ViiA7™ Real-time PCR system (Applied Biosystems, Darmstadt, Germany), as previously reported [79]. To normalize total RNA samples, the small-nuclear-U6 was selected as endogenous control.

The expression of Notch transcript was determined independently by qRT-PCR, using SYBR Green PCR Master Mix. To normalize total RNA samples, GAPDH was selected as an appropriate constitutively expressed endogenous control. The qRT-PCR primer sequences are as follows: Notch, (Forward 5′-GGAGTCAGGGAGAGGTTCTAT-3′ and Reverse 5′-GGAGGTGTGACTAATTGGATGT-3′); GAPDH, (Forward 5′-GGAGTCAACGGATTTGGTCG-3′ and Reverse 5′-CTTCCCGTTCTCAGCCTTGA-3′). The relative expression of the transcripts was measured by using ViiA7™ Real-Time PCR software (Applied Biosystems, Darmstadt, Germany).

### 4.5. Protein Extraction and Western Blot Analysis

CRC and TNBC cell lines transfected with 100 nM miR-34a, miR-34c, and miR-449a mimics or inhibitors, treated or not with AdoMet 500 µM, after 48 h were collected by centrifugation, washed twice with ice-cold PBS, then lysed using 100 µL of RIPA buffer as previously reported [80]. Protein concentration was performed by Bradford method as previously reported [81]. Equal amounts of cell proteins were separated by sodium dodecyl sulfate-polyacrylamide gel electrophoresis (SDS-PAGE) and electrotransferred to nitrocellulose membranes by Trans-Blot turbo Transfer System (Bio-Rad Laboratories, Hercules, CA, USA). The membranes were washed in 10 mM Tris-HCl, pH 8.0, 150 mM NaCl, 0.05% Tween 20 (TBST), and blocked with TBST supplemented with 5% nonfat dry milk. Then, membranes were incubated first with specific primary antibodies at 4 °C overnight in TBST and 5% nonfat dry milk, washed, and then incubated 1 h with HRP-conjugated secondary antibodies. All primary antibodies were used at a dilution of 1:1000; all secondary antibodies were used at a dilution of 1:5000. Blots were then developed using enhanced chemiluminescence detection reagents ECL (Westar, Cyanagen, Bologna, Italy) and exposed to X-ray film. All films were scanned by using Image J software 1.48v (U.S. National Institutes of Health, Bethesda, MD, USA).

### 4.6. Migration Process Evaluated by Scratch-Wound Assay

HCT-116 and Caco-2 cells were seeded in a serum-containing media in 6-well culture plates in the appropriate number and cultured overnight until 100% confluence was reached. The cells were then transfected with 100 nM miR-34a, miR-34c and miR-449a mimics or inhibitors diluted in Opti-MEM free medium integrated or not (Control) with 500 μM AdoMet, by using Lipofectamine 2000 according to manufacturer’s protocol. The wounds were obtained by manually scratching, in a sterile environment, a confluent cell monolayer with a sterile 200 μL pipette tip, rapidly washed twice with medium to remove cell debris and replaced with 2 mL of complete medium. Initial images of the wounds were captured using a microscope (Leica Microsystems GmbH) corresponding to time zero (T0). After 24 h (T1) of treatment, snapshot images were taken to examine the wound closure. Wound areas of control and treated cells were quantified using ImageJ software 1.48v (U.S. National Institutes of Health, Bethesda, MD, USA).

### 4.7. Luciferase Reporter Assay

CRC and TNBC cells were seeded into 24-well plates at 20,000 cells/well and 15,000 cells/well, respectively, and cultured overnight. Thereafter, cells were co-transfected with luciferase pEZX-MT06 plasmids containing wild-type or mutated (5′-GACAATGTCATTTTTC-3′) 3′-UTR of Notch and miR-34a, miR-34c and miR-449a mimics using Lipofectamine 2000, following the provided manual. After 24 h incubation, cell medium was exchanged with fresh DMEM containing 10% FBS. Dual-luciferase reporter assay was conducted using a Dual-Luciferase reporter assay system according to the manufacturer’s instructions. Luciferase activity was detected under the control of Tecan Infinite M200 (Tecan, Männedorf, Switzerland). For the normalization of Firefly luciferase activity, the luminescence intensity of Renilla luciferase was used as an internal control of transfected cells.

### 4.8. Statistical Analysis

Experiments were performed at least three times with replicate samples. Data are expressed as mean ± standard deviation (SD). For the comparisons of mean values between two groups, unpaired Student’s *t*-test was used. For the comparisons of mean values among three or more groups, the means were compared using analysis of variance (ANOVA) plus Bonferroni’s *t*-test. A *p*-value < 0.05 was considered to indicate a statistically significant result, *p* < 0.01 was considered highly significant.

## 5. Conclusions

As a promising tumor suppressor, miR-34s have attracted considerable attention in recent years for their ability to modulate a variety of oncogenic functions in different cancers, and miR-34 mimetics are currently tested for treatment of advanced cancers [82]. However, delivery systems for miR-34 replacement therapy were not without toxicity and side-effects. Notably, the first phase I clinical trial of MRX34, a miR-34a mimic encapsulated in lipid nanoparticles, was stopped because of the occurrence of severe immune-related adverse events [59]. The selective targeting of Notch could also represent an ideal strategy for prevention and treatment of colon cancer owing to the crucial role played by Notch signaling in CRC progression and metastasis. However, despite promising preclinical results and early-phase clinical trials, Notch inhibitors such as anti-Notch receptors/ligands antibodies or γ-secretase inhibitors have failed in clinical translation due to their poor selectivity, dose-limiting toxicity, and low efficacy, encouraging to investigate dietary and natural compounds with multi-target activities to achieve better outcomes [83].

Our study for the first time highlighted the ability of the physiological methyl donor AdoMet to control EMT and the migratory power of CRC cells through the miR-34a/c/miR-449a/Notch axis, thus offering a promising approach for the treatment of this highly invasive cancer. Worthy of interest in this context is to mention that AdoMet is an approved dietary supplement which can therefore be used for therapeutic purposes without the common contraindications of chemotherapy. Moreover, as documented by several clinical studies, AdoMet, at pharmacological doses, shows a low incidence of side-effects with an excellent tolerability record and no toxic or antiproliferative effects in normal, non-tumorigenic cells [21]. The ability of AdoMet to modulate miRNA expression in different cancer cell types and the well-documented potential of miRNA mimics and miRNA inhibitors to restore tumor suppressor miRNAs or to downregulate oncogenic miRNAs, respectively, greatly stimulates the design of adjuvant therapeutic approaches based on combined AdoMet/miRNAs treatments, which could allow synergistic effects resulting in increased cellular chemosensitivity and in reduced drug toxicity.

## Data Availability

The data presented in this study are available on request.

## Supplementary Materials

The following supporting information can be downloaded at: https://www.mdpi.com/article/10.3390/ijms23147673/s1.

## References

[1] Sung H., Ferlay J., Siegel R.L., Laversanne M., Soerjomataram I., Jemal A., Bray F. (2021). Global cancer statistics 2020: GLOBOCAN estimates of incidence and mortality worldwide for 36 cancers in 185 countries. CA A Cancer J. Clin..

[2] Vu T., Datta P.K. (2017). Regulation of EMT in colorectal cancer: A culprit in metastasis. Cancers.

[3] Malki A., ElRuz R.A., Gupta I., Allouch A., Vranic S., Al Moustafa A.E. (2020). Molecular mechanisms of colon cancer progression and metastasis: Recent insights and advancements. Int. J. Mol. Sci..

[4] Tyagi A., Sharma A.K., Damodaran C. (2020). A review on Notch signaling and colorectal cancer. Cells.

[5] Anusewicz D., Orzechowska M., Bednarek A.K. (2021). Notch signaling pathway in cancer-review with bioinformatic analysis. Cancers.

[6] Aster J.C., Pear W.S., Blacklow S.C. (2017). The varied roles of Notch in cancer. Annu. Rev. Pathol. Mech. Dis..

[7] Shaik J.P., Alanazi I.O., Pathan A., Parine N.R., Almadi M.A., Azzam N.A., Aljebreen A.M., Alharbi O., Alanazi M.S., Khan Z. (2020). Frequent activation of Notch signaling pathway in colorectal cancers and its implication in patient survival outcome. J. Oncol..

[8] Allen F., Maillard I. (2021). Therapeutic targeting of Notch signaling: From cancer to inflammatory disorders. Front. Cell. Dev. Biol..

[9] Vinson K.E., George D.C., Fender A.W., Bertrand F.E., Sigounas G. (2016). The Notch pathway in colorectal cancer. Int. J. Cancer.

[10] To K.K., Tong C.W., Wu M., Cho W.C. (2018). MicroRNAs in the prognosis and therapy of colorectal cancer: From bench to bedside. World J. Gastroenterol..

[11] Lewis B.P., Burge C.B., Bartel D.P. (2005). Conserved seed pairing, often flanked by adenosines, indicates that thousands of human genes are microRNA targets. Cell.

[12] Wang W.T., Han C., Sun Y.M., Chen T.Q., Chen Y.Q. (2019). Noncoding RNAs in cancer therapy resistance and targeted drug development. J. Hematol. Oncol..

[13] Si W., Shen J., Zheng H., Fan W. (2019). The role and mechanisms of action of microRNAs in cancer drug resistance. Clin. Epigenetics.

[14] Mollaei H., Safaralizadeh R., Rostami Z. (2019). MicroRNA replacement therapy in cancer. J. Cell. Physiol..

[15] Lai X., Eberhardt M., Schmitz U., Vera J. (2019). Systems biology-based investigation of cooperating microRNAs as monotherapy or adjuvant therapy in cancer. Nucleic Acids Res..

[16] Liu B., Shyr Y., Cai J., Liu Q. (2018). Interplay between miRNAs and host genes and their role in cancer. Brief. Funct. Genom..

[17] Gebert L., MacRae I.J. (2019). Regulation of microRNA function in animals. Nat. Rev. Mol. Cell. Biol..

[18] Ghafouri-Fard S., Glassy M.C., Abak A., Hussen B.M., Niazi V., Taheri M. (2021). The interaction between miRNAs/lncRNAs and Notch pathway in human disorders. Biomed. Pharmacother..

[19] Shen B. (2015). A new golden age of natural products drug discovery. Cell.

[20] Alnuqaydan A.M. (2020). Targeting micro-RNAs by natural products: A novel future therapeutic strategy to combat cancer. Am. J. Transl. Res..

[21] Mosca L., Vitiello F., Pagano M., Coppola A., Veglia Tranchese R., Grillo R., Cacciapuoti G., Porcelli M. (2022). S-adenosylmethionine, a promising antitumor agent in oral and laryngeal cancer. Appl. Sci..

[22] Pascale R.M., Simile M.M., Calvisi D.F., Feo C.F., Feo F. (2022). S-adenosylmethionine: From the discovery of its inhibition of tumorigenesis to its use as a therapeutic agent. Cells.

[23] Ilisso C.P., Sapio L., Delle Cave D., Illiano M., Spina A., Cacciapuoti G., Porcelli M. (2016). S-Adenosylmethionine affects ERK1/2 and Stat3 pathways and induces apoptosis in osteosarcoma cells. J. Cell. Physiol..

[24] Mahmood N., Cheishvili D., Arakelian A., Tanvir I., Khan H.A., Pépin A.S., Szyf M., Rabbani S.A. (2018). Methyl donor S-adenosylmethionine (SAM) supplementation attenuates breast cancer growth, invasion and metastasis in vivo; therapeutic and chemopreventive applications. Oncotarget.

[25] Yan L., Liang X., Huang H., Zhang G., Liu T., Zhang J., Chen Z., Zhang Z., Chen Y. (2019). S-adenosylmethionine affects cell cycle pathways and suppresses proliferation in liver cells. J. Cancer.

[26] Mosca L., Minopoli M., Pagano M., Vitiello F., Carriero M.V., Cacciapuoti G., Porcelli M. (2020). Effects of S-adenosyl-l-methionine on the invasion and migration of head and neck squamous cancer cells and analysis of the underlying mechanisms. Int. J. Oncol..

[27] Mahmood N., Arakelian A., Muller W.J., Szyf M., Rabbani S.A. (2020). An enhanced chemopreventive effect of methyl donor S-adenosylmethionine in combination with 25-hydroxyvitamin D in blocking mammary tumor growth and metastasis. Bone Res..

[28] Mahmood N., Arakelian A., Cheishvili D., Szyf M., Rabbani S.A. (2020). S-adenosylmethionine in combination with decitabine shows enhanced anti-cancer effects in repressing breast cancer growth and metastasis. J. Cell. Mol. Med..

[29] Mosca L., Pagano M., Ilisso C.P., Delle Cave D., Desiderio V., Mele L., Caraglia M., Cacciapuoti G., Porcelli M. (2019). AdoMet triggers apoptosis in head and neck squamous cancer by inducing ER-stress and potentiates cell sensitivity to cisplatin. J. Cell. Physiol..

[30] Cave D.D., Desiderio V., Mosca L., Ilisso C.P., Mele L., Caraglia M., Cacciapuoti G., Porcelli M. (2017). S-Adenosylmethionine-mediated apoptosis is potentiated by autophagy inhibition induced by chloroquine in human breast cancer cells. J. Cell. Physiol..

[31] Mosca L., Vitiello F., Coppola A., Borzacchiello L., Ilisso C.P., Pagano M., Caraglia M., Cacciapuoti G., Porcelli M. (2020). Therapeutic potential of the natural compound S-adenosylmethionine as a chemoprotective synergistic agent in breast, and head and neck cancer treatment: Current status of research. Int. J. Mol. Sci..

[32] Ilisso C.P., Delle Cave D., Mosca L., Pagano M., Coppola A., Mele L., Caraglia M., Cacciapuoti G., Porcelli M. (2018). Adenosylmethionine regulates apoptosis and autophagy in MCF-7 breast cancer cells through the modulation of specific microRNAs. Cancer Cell Int..

[33] Coppola A., Ilisso C.P., Stellavato A., Schiraldi C., Caraglia M., Mosca L., Cacciapuoti G., Porcelli M. (2020). S-Adenosylmethionine inhibits cell growth and migration of triple negative breast cancer cells through upregulating MiRNA-34c and MiRNA-449a. Int. J. Mol. Sci..

[34] Pagano M., Mosca L., Vitiello F., Ilisso C.P., Coppola A., Borzacchiello L., Mele L., Caruso F.P., Ceccarelli M., Caraglia M. (2020). Mi-RNA-888-5p is involved in S-adenosylmethionine antitumor effects in laryngeal squamous cancer cells. Cancers.

[35] Mosca L., Vitiello F., Borzacchiello L., Coppola A., Tranchese R.V., Pagano M., Caraglia M., Cacciapuoti G., Porcelli M. (2021). Mutual correlation between non-coding RNA and S-adenosylmethionine in human cancer: Roles and therapeutic opportunities. Cancers.

[36] Mosca L., Pagano M., Pecoraro A., Borzacchiello L., Mele L., Cacciapuoti G., Porcelli M., Russo G., Russo A. (2020). S-adenosyl-L-methionine overcomes uL3-mediated drug resistance in p53 deleted colon cancercells. Int. J. Mol. Sci..

[37] Mosca L., Pagano M., Borzacchiello L., Mele L., Russo A., Russo G., Cacciapuoti G., Porcelli M. (2021). S-adenosylmethionine increases the sensitivity of human colorectal cancer cells to 5-fluorouracil by inhibiting P-glycoprotein expression and NF-B activation. Int. J. Mol. Sci..

[38] Zsigrai S., Kalmár A., Nagy Z.B., Barták B.K., Valcz G., Szigeti K.A., Galamb O., Dankó T., Sebestyén A., Barna G. (2020). S-adenosylmethionine treatment of colorectal cancer cell lines alters DNA methylation, DNA repair and tumor progression-related gene expression. Cells.

[39] Zhang N., Hu X., Du Y., Du J. (2021). The role of miRNAs in colorectal cancer progression and chemoradiotherapy. Biomed. Pharmacother..

[40] Niki M., Nakajima K., Ishikawa D., Nishida J., Ishifune C., Tsukumo S.I., Shimada M., Nagahiro S., Mitamura Y., Yasutomo K. (2017). MicroRNA-449a deficiency promotes colon carcinogenesis. Sci. Rep..

[41] Ishikawa D., Takasu C., Kashihara H., Nishi M., Tokunaga T., Higashijima J., Yoshikawa K., Yasutomo K., Shimada M. (2019). The significance of MicroRNA-449a and its potential target HDAC1 in patients with colorectal cancer. Anticancer Res..

[42] Veena M.S., Raychaudhuri S., Basak S.K., Venkatesan N., Kumar P., Biswas R., Chakrabarti R., Lu J., Su T., Gallagher-Jones M. (2020). Dysregulation of hsa-miR-34a and hsa-miR-449a leads to overexpression of PACS-1 and loss of DNA damage response (DDR) in cervical cancer. J. Biol. Chem..

[43] Zhang L., Liao Y., Tang L. (2019). MicroRNA-34 family: A potential tumor suppressor and therapeutic candidate in cancer. Exp. Clin. Cancer Res..

[44] Luo W., Huang B., Li Z., Li H., Sun L., Zhang Q., Qiu X., Wang E. (2013). MicroRNA-449a is downregulated in non-small cell lung cancer and inhibits migration and invasion by targeting c-Met. PLoS ONE.

[45] Sandbothe M., Buurman R., Reich N., Greiwe L., Vajen B., Gürlevik E., Schäffer V., Eilers M., Kühnel F., Vaquero A. (2017). The microRNA-449 family inhibits TGF-β-mediated liver cancer cell migration by targeting SOX4. J. Hepatol..

[46] Gialeli C., Theocharis A.D., Karamanos N.K. (2011). Roles of matrix metalloproteinases in cancer progression and their pharmacological targeting. FEBS J..

[47] Salem N., Kamal I., Al-Maghrabi J., Abuzenadah A., Peer-Zada A.A., Qari Y., Al-Ahwal M., Al-Qahtani M., Buhmeida A. (2016). High expression of matrix metalloproteinases: MMP-2 and MMP-9 predicts poor survival outcome in colorectal carcinoma. Future Oncol..

[48] Loh C.Y., Chai J.Y., Tang T.F., Wong W.F., Sethi G., Shanmugam M.K., Chong P.P., Looi C.Y. (2019). The E-cadherin and N-cadherin switch in epithelial-to-mesenchymal transition: Signaling, therapeutic implications, and challenges. Cells.

[49] Strouhalova K., Přechová M., Gandalovičová A., Brábek J., Gregor M., Rosel D. (2020). Vimentin intermediate filaments as potential target for cancer treatment. Cancers.

[50] Bian J., Dannappel M., Wan C., Firestein R. (2020). Transcriptional regulation of Wnt/β-catenin pathway in colorectal cancer. Cells.

[51] Agarwal V., Bell G.W., Nam J., Bartel D.P. (2015). Predicting effective microRNA target sites in mammalianm RNAs. eLife.

[52] Kozomara A., Birgaoanu M., Griffths-Jones S. (2019). miRBase: From microRNA sequences to function. Nucleic Acids Res..

[53] Xiu M.X., Liu Y.M. (2019). The role of oncogenic Notch2 signaling in cancer: A novel therapeutic target. Am. J. Cancer Res..

[54] Niu L., Yang W., Duan L., Wang X., Li Y., Xu C., Liu C., Zhang Y., Zhou W., Liu J. (2020). Biological implications and clinical potential of metastasis-related miRNA in colorectal cancer. Mol. Ther.-Nucleic Acids.

[55] Lu S.C. (2000). S-adenosylmethionine. Int. J. Biochem. Cell Biol..

[56] Lu S.C., Mato J.M. (2008). S-adenosylmethionine in cell growth, apoptosis and liver cancer. J. Gastroenterol. Hepatol..

[57] Lu S.C., Mato J.M. (2012). S-adenosylmethionine in liver health, injury, and cancer. Physiol. Rev..

[58] Lv J., Zhang Z., Pan L., Zhang Y. (2019). MicroRNA-34/449 family and viral infections. Virus Res..

[59] Li W.J., Wang Y., Liu R., Kasinski A.L., Shen H., Slack F.J., Tang D.G. (2021). MicroRNA-34a: Potent tumor suppressor, cancer stem cell inhibitor, and potential anticancer therapeutic. Front. Cell Dev. Biol..

[60] Bommer G.T., Gerin I., Feng Y., Kaczorowski A.J., Kuick R., Love R.E., Zhai Y., Giordano T.J., Qin Z.S., Moore B.B. (2007). p53-mediated activation of miRNA34 candidate tumor-suppressor genes. Curr. Biol..

[61] Rokavec M., Li H., Jiang L., Hermeking H. (2014). The p53/miR-34 axis in development and disease. J. Mol. Cell Biol..

[62] Okada N., Lin C.P., Ribeiro M.C., Biton A., Lai G., He X., Bu P., Vogel H., Jablons D.M., Keller A.C. (2014). A positive feedback between p53 and miR-34 miRNAs mediates tumor suppression. Genes Dev..

[63] Toyota M., Suzuki H., Sasaki Y., Maruyama R., Imai K., Shinomura Y., Tokino T. (2008). Epigenetic silencing of microRNA-34b/c and B-cell translocation gene 4 is associated with CpG island methylation in colorectal cancer. Cancer Res..

[64] Sun X., Liu S., Chen P., Fu D., Hou Y., Hu J., Liu Z., Jiang Y., Cao X., Cheng C. (2016). miR-449a inhibits colorectal cancer progression by targeting SATB2. Oncotarget.

[65] Lan S.H., Lin S.C., Wang W.C., Yang Y.C., Lee J.C., Lin P.W., Chu M.L., Lan K.Y., Zuchini R., Liu H.S. (2021). Autophagy upregulates miR-449a expression to suppress progression of colorectal cancer. Front. Oncol..

[66] Herszényi L., Hritz I., Lakatos G., Varga M.Z., Tulassay Z. (2012). The behavior of matrix metalloproteinases and their inhibitors in colorectal cancer. Int. J. Mol. Sci..

[67] Wang Q., Zhu G., Lin C., Lin P., Chen H., He R., Huang Y., Yang S., Ye J. (2021). Vimentin affects colorectal cancer proliferation, invasion, and migration via regulated by activator protein 1. J. Cell. Phys..

[68] Cancer Genome Atlas Network (2012). Comprehensive molecular characterization of human colon and rectal cancer. Nature.

[69] Zhan T., Rindtorff N., Boutros M. (2017). Wnt signaling in cancer. Oncogene.

[70] Ye K., Xu C., Hui T. (2019). MiR-34b inhibits the proliferation and promotes apoptosis in colon cancer cells by targeting Wnt/β-catenin signaling pathway. Biosci. Rep..

[71] Cheng X., Xu X., Chen D., Zhao F., Wang W. (2019). Therapeutic potential of targeting the Wnt/β-catenin signaling pathway in colorectal cancer. Biomed. Pharmacother..

[72] Yu W.K., Xu Z.Y., Yuan L., Mo S., Xu B., Cheng X.D., Qin J.J. (2020). Targeting β-catenin signaling by natural products for cancer prevention and therapy. Front. Pharmacol..

[73] Majidinia M., Darband S.G., Kaviani M., Nabavi S.M., Jahanban-Esfahlan R., Yousefi B. (2018). Cross-regulation between Notch signaling pathway and miRNA machinery in cancer. DNA Repair.

[74] Jia Y., Lin R., Jin H., Si L., Jian W., Yu Q., Yang S. (2019). MicroRNA-34 suppresses proliferation of human ovarian cancer cells by triggering autophagy and apoptosis and inhibits cell invasion by targeting Notch 1. Biochimie.

[75] Zhang X., Ai F., Li X., Tian L., Wang X., Shen S., Liu F. (2017). MicroRNA-34a suppresses colorectal cancer metastasis by regulating Notch signaling. Oncol. Lett..

[76] Xia J., Duan Q., Ahmad A., Bao B., Banerjee S., Shi Y., Ma J., Geng J., Chen Z., Rahman K.M. (2012). Genistein inhibits cell growth and induces apoptosis through up-regulation of miR-34a in pancreatic cancer cells. Curr. Drug Targets.

[77] Bauer S., Ratz L., Heckmann-Nötzel D., Kaczorowski A., Hohenfellner M., Kristiansen G., Duensing S., Altevogt P., Klauck S.M., Sültmann H. (2021). miR-449a repression leads to enhanced Notch signaling in *TMPRSS2: ERG* fusion positive prostate cancer cells. Cancers.

[78] Mercey O., Popa A., Cavard A., Paquet A., Chevalier B., Pons N., Magnone V., Zangari J., Brest P., Zaragosi L.E. (2017). Characterizing isomiR variants within the microRNA-34/449 family. FEBS Lett..

[79] Stasio D.D., Mosca L., Lucchese A., Cave D.D., Kawasaki H., Lombardi A., Porcelli M., Caraglia M. (2019). Salivary mir-27b expression in oral lichen planus patients: A series of cases and a narrative review of literature. Curr. Top. Med. Chem..

[80] Mele L., Del Vecchio V., Marampon F., Regad T., Wagner S., Mosca L., Bimonte S., Giudice A., Liccardo D., Prisco C. (2020). β2-AR blockade potentiates MEK1/2 inhibitor effect on HNSCC by regulating the Nrf2-mediated defense mechanism. Cell Death Dis..

[81] Minici C., Mosca L., Ilisso C.P., Cacciapuoti G., Porcelli M., Degano M. (2020). Structures of catalytic cycle intermediates of the *Pyrococcus furiosus* methionine adenosyltransferase demonstrate negative cooperativity in the archaeal orthologues. J. Struct. Biol..

[82] Adams B.D., Parsons C., Slack F.J. (2016). The tumor-suppressive and potential therapeutic functions of miR-34a in epithelial carcinomas. Expert Opin. Ther. Targets.

[83] Kiesel V.A., Stan S.D. (2022). Modulation of Notch signaling pathway by bioactive dietary agents. Int. J. Mol. Sci..

