# Supplementary material for: S-Adenosylmethionine Inhibits Colorectal Cancer Cell Migration through Mirna-Mediated Targeting of Notch Signaling Pathway
Source: Int J Mol Sci. 2022 Jul 12;23(14):7673. doi: 10.3390/ijms23147673 (PMC9320859; doi:10.3390/ijms23147673)
Supplement: Supplementary file 1 [file ijms-23-07673-s001.zip › ijms-1808985-supplementary.pdf]

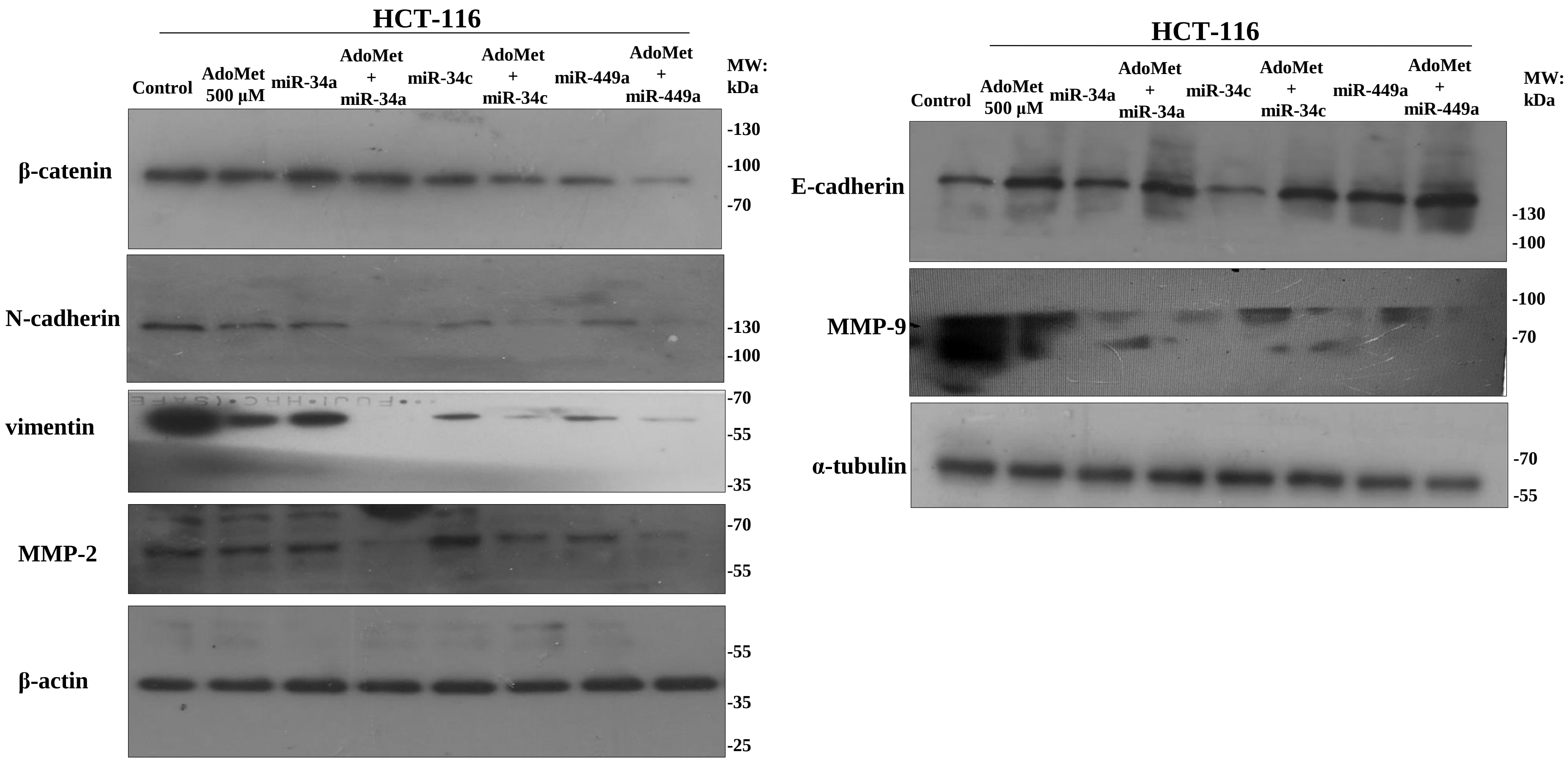

Figure S1: Effect of AdoMet alone and in combination with miR-34a, miR-34c, and miR-449a mimics on the levels of migration- and EMT-related proteins in HCT-116 cells. The cropped blots are used in the main Figure (Figure 3).

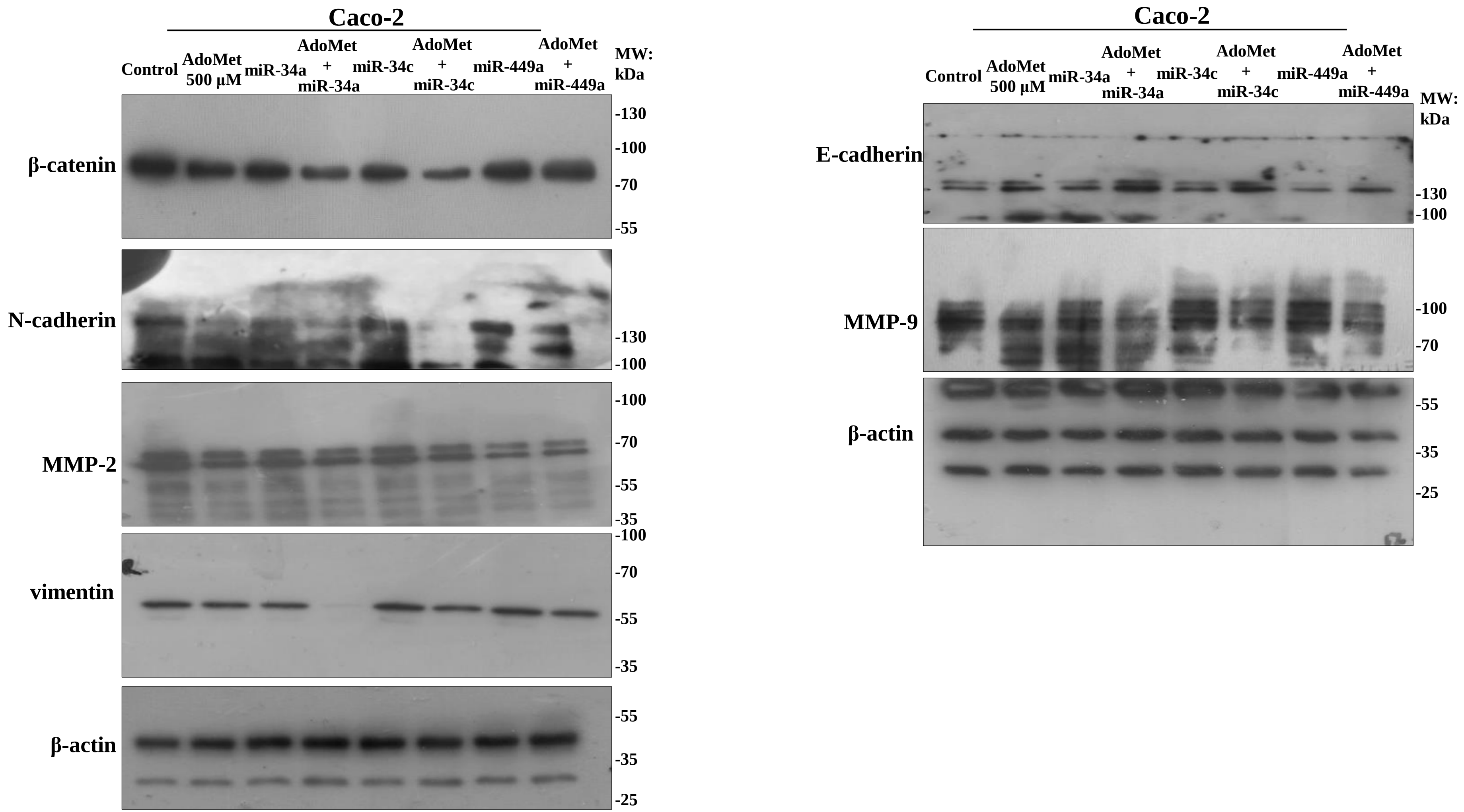

Figure S2: Effect of AdoMet alone and in combination with miR-34a, miR-34c, and miR-449a mimics on the levels of migration- and EMT-related proteins in Caco-2 cells. The cropped blots are used in the main Figure (Figure 3).

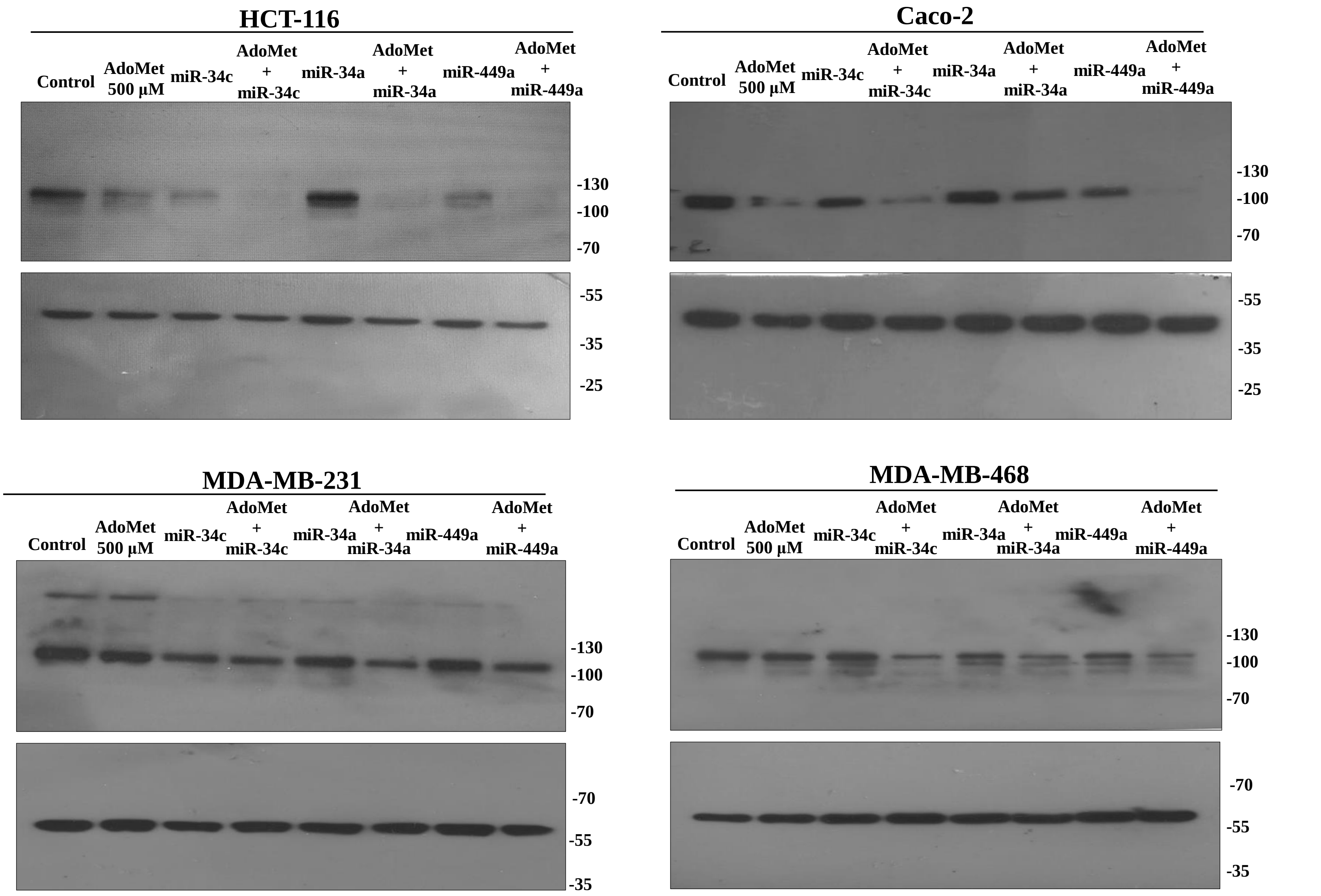

Figure S3: Effect of AdoMet and miR-34a, miR-34c, and miR-449a mimics on Notch protein levels in CRC and TNBC cells. The cropped blots are used in the main Figure (Figure 6).

Table S1. Effect of AdoMet on cell migration- and EMT-related protein expression in CRC and TNBC* cell lines.

Protein	Effect of AdoMet	Biological function
MMP-2	down-regulation	cell migration ↓
MMP-9	down-regulation	cell migration ↓
E-cadherin	up-regulation	EMT, cell migration ↓
N-cadherin	down-regulation	EMT, cell migration ↓
vimentin	down-regulation	EMT, cell migration ↓
β-catenin	down-regulation	cell growth and invasion ↓
Notch	down-regulation	tumor development and metastasis ↓

* Referred to [33].

Explanatory notes: ↓ decrease
